# Neurodevelopmental Disorders and Adaptive Functions: A Study of Children With Autism Spectrum Disorders (ASD) and/or Attention Deficit and Hyperactivity Disorder (ADHD)

**DOI:** 10.3389/fpsyt.2019.00673

**Published:** 2019-09-04

**Authors:** Valeria Scandurra, Leonardo Emberti Gialloreti, Francesca Barbanera, Marirosa Rosaria Scordo, Angelo Pierini, Roberto Canitano

**Affiliations:** ^1^Division of Child and Adolescent Neuropsychiatry, University Hospital of Siena, Siena, Italy; ^2^Department of Biomedicine and Prevention, University of Rome Tor Vergata, Rome, Italy; ^3^Department of Child Neuropsychiatry, ASL Umbria 1, Perugia, Italy; ^4^Department of Child Neuropsychiatry, Meyer University Hospital, Firenze, Italy

**Keywords:** autism spectrum disorders (ASD), attention deficit hyperactivity disorder (ADHD), adaptive function, children, comorbidity

## Abstract

**Introduction**: Autism spectrum disorder (ASD) and attention deficit and hyperactivity disorder (ADHD) are the two most common neurodevelopmental disorders observed in childhood. The DSM-5 accepts a combined diagnosis of ADHD and ASD, while the DSM-IV did not. The aim of this study was to identify and evaluate the adaptive profile of children and adolescents with a diagnosis of comorbid ADHD and ASD, in comparison with adaptive functioning in subjects with a diagnosis of only ASD or ADHD.

**Materials and Methods:** Ninety-one children (77 boys, 14 girls), aging from 3.1 to 13.4 years (mean age: 8.3 ± 7.2), who met the criteria for a diagnosis of ASD and/or ADHD were enrolled. A neuropsychological evaluation involving cognitive and adaptive assessment was conducted using the Autism Diagnostic Observation Schedule – Second Edition (ADOS-2), the Conners’ Parent Rating Scale – Revised: Long Version (CPRS-R), the Wechsler Intelligence Scale – Fourth Edition or the Griffiths Mental Developmental Scales – Extended Revised, the Vineland Adaptive Behaviour Scale – Second Edition (VABS-II).

**Conclusion:** As to the adaptive skills in the three groups evaluated, a worse general profile was ascertained in the ASD and in ASD plus ADHD groups in comparison with respect to the ADHD-only group. With VABS-II evaluation, we found significant differences among the three groups across all domains and combined scores: Communication (F = 18.960; p < 0.001), Socialization (F = 25.410; p < 0.001), Daily Living Skills (F = 19.760; p < 0.001), Motor (F = 9.615; p < 0.001), and Adaptive behavior composite [ABC] (F = 29.370; p < 0.001). Implications of neurodevelopmental double diagnosis such as ASD plus ADHD are discussed.

## Introduction

Autism spectrum disorder (ASD) and attention deficit and hyperactivity disorder (ADHD) are the two most common neurodevelopmental disorders observed in childhood (1, 2). According to the Diagnostic and Statistical Manual of Mental Disorders, 5th edition (DSM-5) (1), ASD is a disorder characterized by deficits in social communication as well as restricted, repetitive patterns of behaviors. In the child population, ASD prevalence has been estimated to be about 1%, but a recently published US study put it at 1.68% (3). Approximately 30% of children with ASD undergo a regression of development with variable course that maybe associated with epileptic abnormalities in an undetermined percentage (4, 5).

Attention-deficit/hyperactivity disorder (ADHD) is one of the most common mental disorders affecting children. Symptoms of ADHD include inattention, hyperactivity, and impulsivity. Current estimated prevalence is 5% of children and 2.5% in adults. ADHD is often first identified in school-aged children when it leads to disruption in the classroom and/or difficulties with school duties. It is more common among boys than girls (DSM-5) (1).

Impairment of social competences in neurodevelopmental disorders is common and needs to be thoroughly addressed. In recent years, there has been increasing interest in the diagnostic overlap and similarities between ADHD and ASD (6–11). Evidence indicated that both disorders co-occur with a high frequency, in 20–50% of children with ADHD meeting criteria for ASD and in 30–80% of ASD children meeting criteria for ADHD. (12). The co-occurrence of ASD and ADHD was found to increases with age, appearing in school age children more clearly, with severity of ASD and ADHD symptoms and with lower IQ (8, 13). Moreover, the increase in inattention and impulsivity concomitant with increases in ASD severity may be able to predict the severity of challenging behaviors and social skills deficits in toddlers and should be carefully evaluated in this population (14–16). In addition individuals with ASD frequently experience additional psychiatric comorbiditties (17).

As to adaptive functions, some studies found that children with ASD+ADHD showed a more severe impairment in adaptive functioning and a poorer quality of life than children with ASD alone (18) while other studies found varying profiles depending on cognitive level and age (19, 20).

A specific social-communication core deficit, associated with restricted and repetitive behaviors (RRBs) is the hallmark of ASD. In contrast to the DSM-IV (APA 1994), the DSM-5 (1) allows a combined diagnosis of ADHD and ASD. Both ASD and ADHD develop from interactions among multiple genetic and environmental factors, which have an effect on complex neurobiological systems already during prenatal life. These interactions are likely to be involved in the distinct developmental trajectories, clinical characteristics, and outcomes of the two disorders (21).

Children with ADHD frequently display peculiar social difficulties. Social competences in ADHD are thought to be related to self-regulation difficulties, low social skills adaptive level, and attentional issues, which can impact the overall ability to process social information. Children diagnosed with predominantly inattentive ADHD (PI) are more passive, less aggressive, less assertive, and less knowledgeable of appropriate social behavior than those diagnosed with combined ADHD (CB). Children with PI are more likely than typical children to be socially neglected, whereas children with CB are more likely to be socially rejected (6). Children with ADHD may have low social impact; their isolation and/or intrusive approaches to other children can be mistaken for unawareness of social rules, as in ASD (22). At the neuropsychological level, both ADHD and ASD present difficulties in executive function (EF), even if EF deficits might differ between the two disorders. Inhibitory dysfunction is characteristic of ADHD, while in ASD central coherence and theory of mind deficits play a major role (23–25). Studies investigating the potential influence of ADHD on ASD have reported contrasting results regarding its influence on autistic symptoms severity (26, 27). Furthermore, several studies have noted that children suffering from both disorders generally present a more severe psychiatric burden. It was observed that children with both ASD and ADHD were more likely to have conduct problems or anxiety or depression symptoms than children with ASD only (28–30). Neurophysiological investigation using event-related potentials (ERPs) on these conditions detected a dissociation between disorders on the basis of distinct stages of emotion processing (31, 32). Further investigations demonstrated that children with ASD and ASD + ADHD showed reduced theta and alpha power on quantitative electroencephalographic studies compared to children without ASD e.g. controls and ADHD (33).

In research on interventions, children with ASD+ADHD undergoing social skills training failed to improve, as opposed to children with ASD only and children with ASD and anxiety disorder (34).

The main aim of this study was to identify and evaluate the adaptive functions of children and adolescents with a diagnosis of ASD+ADHD, in comparison with adaptive functioning in subjects with a diagnosis of ASD or ADHD.

## Participants

This cross-sectional study included 91 children (77 boys, 14 girls), ranging from 3.1 to 13.4 years (mean age: 8.3 ± 7.2), who met the criteria for a diagnosis of ASD and/or ADHD (**Table 1**). The children were consecutively recruited between January 2016 and December 2017 among those referred to our outpatient clinic for assessment and diagnosis of developmental difficulties, and were enrolled in the study according to the clinical features that were ascertained during evaluation and that pointed to the mentioned diagnostic domains.

All children underwent a full clinical evaluation, including medical history, clinical observations, and assessment. Diagnosis of ASD was based on DSM-5 criteria. Impairments in intentional communication, eye contact engagement, shared attention behavior, use of gestures, and restrictive and repetitive behaviors were assessed and detailed. Diagnoses were also confirmed by using the Autism Diagnostic Observation Schedule – Second Edition (ADOS-2) (35).

**Table 1:**
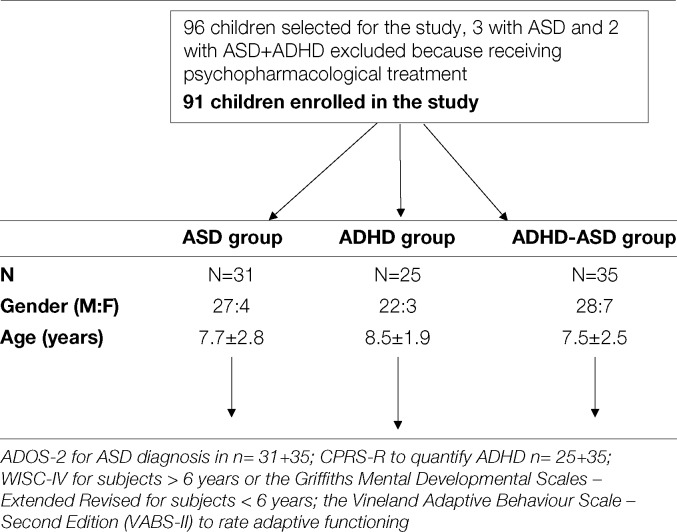
Flow chart of the protocol of the study.

Diagnosis of ADHD was based on DSM-5 criteria. Overall, clinical criteria evaluation contributed to defining the diagnostic classification. In addition to the ADHD features of hyperactivity, impulsivity, and/or inattentive problems, relevant social deficits – if present – were also thoroughly described. We also used the Conners’ Parent Rating Scale – Revised: Long Version to confirm the ADHD diagnoses n = 25 (CPRS-R) (Conners 2001).

Exclusion criteria included (1) genetic and neurodevelopmental disorders of known etiology (such as tuberous Sclerosis, fragile X syndrome, and chromosomal abnormalities), (2) serious chronic diseases, (3) significant sensory or motor impairment, (4) presence of seizures, and (5) use of psychoactive drugs as possibly interfering with the clinical profileAccording to the diagnostic criteria, 31 children have been identified as ASD only (ASD group), 25 as ADHD only (ADHD group), and 35 as ADHD and ASD (ASD+ADHD group). Characteristics of the groups are described in **Table 2**.

**Table 2:** Demographic and clinical characteristics of the study groups.

	ASD group	ADHD group	ADHD-ASD group	p
**N**	31	25	35	
**Gender (M:F)**	27:4	22:3	28:7	0.625
**Age (years)**	7.7 ± 2.8	8.5 ± 1.9	7.5 ± 2.5	0.300
**IQ**	81.4 ± 18.7	103.3 ± 17.0	76.5 ± 20.6	<0.001
				
**VABS-II**				
**Socialization**	71.5 ± 11.2	88.4 ± 7.5	68.6 ± 13.0	<0.001
**Communicativon**	75.3 ± 11.8	87.3 ± 8.7	71.0 ± 10.0	<0.001
**Daily living skills**	73.6 ± 8.4	89.4 ± 12.8	70.9 ± 13.6	<0.001
**Motor skills**	84.5 ± 11.7	95.3 ± 12.1	80.7 ± 14.4	<0.001
**ABC**	71.9 ± 8.2	86.8 ± 8.0	69.4 ± 10.6	<0.001
				
**CPRS-R (ADHD Index)**				
**PI**		74.3 ± 10.5	78.4 ± 10.7	0.163
**PH**		68.7 ± 13.2	68.2 ± 12.0	0.893
**CB**		73.9 ± 12.0	75.6 ± 9.6	0.631
**PI : CB**		5:20	11:24	0.324
				
**ADOS-2**				
**ADOS value**	16.5 ± 4.9		15.2 ± 4.5	0.244
**CSS (comparison severity scores)**	7.8 ± 1.7		7.6 ± 1.6	0.622

Quantitative variables presented as mean ± standard deviation. VABS-II, Vineland Adaptive Behaviour Scale, second edition; CPRS-R: Conners’ Parent Rating Scale-Revised: Long Version; ADOS-2: Autism Diagnostic Observation Schedule- Second Edition. ABC, Adaptive Behaviour Composite; PI, Prevalent inattentive clinical presentation; PH, Predominantly Hyperactivity/Impulsivity clinical presentation; CB, Combined clinical presentation; CSS, Calibrated Severity Score. Quantitative variables presented as mean ± standard deviation.

After complete description of the study to parents and/or guardians, written informed consent for data acquisition and clinical examination was obtained according to procedures approved by the local ethics committee. The research was conducted according to the rules of the Helsinki Declaration regarding good clinical practice and ethics.

The study was approved by our local ethics committee.

## Measures

All children were examined by our research team. The neuropsychological evaluation involving cognitive and adaptive assessment was conducted by means of a diagnostic protocol in order to identify the main clinical and developmental features and to depict a comprehensive profile of the children enrolled in the study. The protocol included the administration of (1) the ADOS-2 for measuring the severity of autistic symptoms (35) to children with ASD symptoms n = 31 + 35; (2) the CPRS-R to quantify the severity of ADHD symptoms (Conners 2001); (3) the Wechsler Intelligence Scale – Fourth Edition (WISC-IV) (36) for subjects >6 years or the Griffiths Mental Developmental Scales – Extended Revised (GMDS-ER; Griffiths) for subjects < 6 years to evaluate intellectual functioning; (4) the Vineland Adaptive Behaviour Scale – Second Edition (VABS-II) (37) to rate adaptive functioning.

The ADOS-2 was administered by experienced and board certified examiners to determine the severity of ASD symptoms. It is a semi-structured, standardized assessment instrument designed to obtain information about social-communication development and repetitive and restricted interests in children. This tool is considered the gold standard for ASD evaluation and is widely used in clinical practice. The ADOS-2 diagnostic algorithm yields classifications of ASD versus non-ASD children and a calibrated severity score (CSS) for the algorithm total that provides further information, including the severity of the disorder.

The CPRS-R is a tool for obtaining parental reports of childhood behavior problems that contains summary scales supporting ADHD diagnosis and quantifying ADH severity. This scale has a seven-factor model composed of the following factors: Cognitive Problems, Oppositional, Hyperactivity–Impulsivity, Anxious–Shy, Perfectionism, Social Problems, and Psychosomatic. It has good internal reliability, high test–retest reliability, and effective discriminatory power. In addition, the CPRS-R includes a corresponding factor structure with the Conners Teacher Rating Scale – Revised and comprehensive symptom coverage for ADHD and related disorders. Three types of ADHD are now recognized: predominantly inattentive (PI), predominantly hyperactive–impulsive (PH) and combined (CB).

To evaluate intellectual functioning and determine the IQ, we administered – according to the age of the child – the WISC-IV or the GMDS-ER. Both scales provide a value that represents the subject’s general intellectual ability. These measures are standardized by chronological age, with a mean of 100 ± 15. In this study, we considered IQ indicators to be the Full Scale Intellectual Quotient (FSIQ) for the WISC-IV and the developmental quotient (DQ) for the Griffiths Scales.

The VABS-II is a semi-structured parent interview used to obtain parent ratings of children’s adaptive functioning across three domains: Communication, Socialization, and Daily Living skills. Standard scores were obtained for each domain and combined to provide an Adaptive behaviour composite (ABC) standard score. VABS-II scores have a mean of 100 ± 15, with lower scores indicating more severe impairment.

## Statistical Analysis

Comparisons between groups were examined, as appropriate, by means of one-way analysis of variance (ANOVA) followed by post-hoc Welch two-sample t-test and Tukey contrasts for multiple comparisons of means, as well as by means of Pearson’s chi-squared test, Kruskal-Wallis rank sum test, and Wilcoxon rank sum test with continuity correction. Linear regression model with ABC as outcome variable was used to model several covariates. Variables were entered according to a procedure of forward selection. The first variable entered into the equation was the one with the largest correlation with ABC. The next variables were added according to the largest change in the R 2 statistic, until no more change occurred. The chosen model was the one with the largest R2. Goodness-of-fit statistics are shown: Multiple R 2, Adjusted R 2,

F-statistics, standard error of the estimate, and p-value. An alpha level of 0.05 was used for all statistical analyses. Results, if not otherwise specified, are given as means ± SDs. All statistical analyses were performed using the *R* Language and Environment for Statistical Computing program (http://www.R-project.org; accessed October 2018).

## Results

As shown in **Table 2**, mean age and gender ratio did not differ between the three groups (F = 1.221; p = 0.300 and χ^2^ = 0.939; p = 0.625). IQ was different among groups (F = 15.140; p < 0.001). The post-hoc analysis showed that IQ was significantly higher in the ADHD group, compared to the ASD (t = 4.232; p < 0.001) or to the ASD+ADHD group (t = 5.317; p < 0.001), while there was no significant difference between the ASD and the ASD+ADHD group (t = 1.048; p = 548).

In terms of parent ratings of children’s adaptive functioning, measured by means of the VABS-II, we found significant differences among the three groups across all domains and combined scores: Communication (F = 18.960; p < 0.001), Socialization (F = 25.410; p < 0.001), Daily Living Skills (F = 19.760; p < 0.001), Motor (F = 9.615; p < 0.001), and ABC (F = 29.370; p < 0.001) (**Table 3**). Subsequent post-hoc analyses showed that higher mean scores always depended on ADHD either alone or combined, while there were no statistically significant differences between the groups that presented with ASD.

**Table 3:** *Post-hoc* analyses for VABS-II values.

Domains	ASD vs. ADHD		ASD+ADHD vs. ADHD		ASD+ADHD vs. ASD	
	t	p	t	P	t	p
**Socialization**	5.663	<0.001	6.782	<0.001	1.028	=0.561
**Communication**	4.344	<0.001	6.061	<0.001	1.700	=0.211
**Daily living skills**	4.969	<0.001	5.992	<0.001	0.946	=0.612
**Motor skills**	3.067	<0.01	4.314	<0.001	1.165	=0.477
**ABC**	6.086	<0.001	7.291	<0.001	1.108	=0.512

VABS-II, Vineland Adaptive Behaviour Scale, second edition; ABC, Adaptive Behaviour Composite.

Considering clinical presentations in the two groups presenting ADHD features, in the ADHD-only group we observed 20 CB and 5 PI presentations; in the ASD+ADHD group, 24 CB and 11 PI presentations. The difference between the two groups was not statistically significant (χ^2^ = 0.974; p = 0.324).

In terms of ADHD symptom severity, the mean score of the Conners Index was 76.7 ± 7.2 in the ADHD group and 79.6 ± 10.9 in the ASD+ADHD group. The difference was not statistically significant (W = 376; p = 0.360). Overall, none of Conners indexes showed relevant differences between the ASD+ADHD and the ADHD-only group (**Table 2**).

ADOS-2 total scores were similar in the two groups with ASD features (ASD group: 16.5 ± 4.9; ASD+ADHD group: 15.1 ± 4.5) with no statistically significant differences between the two groups (t = 1.177; p = 0.244).

In a linear regression model, higher VABS-II ABC scores were negatively associated with age and ASD diagnosis, and positively associated with IQ. There was no significant association with gender (**Table 4**).

**Table 4:** Linear Regression Model. Outcome variable: Adaptive Behaviour Composite (ABC) of the VABS-II.

	Beta	SE	t	p
(Intercept)	77.567	5.952	13.032	<0.001
**Age**	–0.871	0.373	–2.336	<0.05
**ASD diagnosis**	–12.387	2.533	–4.890	<0.001
**ASD+ADHD diagnosis**	–14.218	2.592	–5.484	<0.001
**IQ**	0.164	0.048	3.453	<0.001
**Gender**	0.487	2.723	0.179	=0.858

Overall model: p < 0.001, Multiple R2: 0.505; Adjusted R2: 0.482. F-statistics: 21.7; Residual standard error: 8.46 on 85 degrees of freedom (1 observation missing). SE, Standard Error; VABS-II, Vineland Adaptive Behaviour Scale, second edition.

## Discussion

Research focusing on co-occurring ADHD and ASD has been directed primarily on origins and clinical characteristics and with minor effort on interventions. Children with ADHD and ASD experience more difficulty in daily situations as compared to those with only one disorder. Co-occurrence of ADHD and ASD is associated with a lower quality of life and poorer adaptive functioning as compared to children with ASD only (38). In addition, co-occurring ADHD and ASD may be less responsive to standard treatments for either disorder than individuals with only one form of the disorders. At present there are few reports regarding developmental trajectories when ADHD and ASD co-occur and it may be important to examine whether early ASD treatment can influence the stability of ADHD symptoms and vice versa (22).

As to the adaptive skills, in the current study a worse general profile was ascertained in ASD and in ASD+ADHD groups with respect to the ADHD-only group in all VABS-II domains (Communication, Daily Living Skills, and Socialization) and ABC. Slightly lower scores not statistically significant were found in the combined group with ASD+ADHD in comparison to ASD group detected with respect to all the VABS-II domains: Communication, Daily Living Skills, and Socialization (37).

Other studies compared adaptive profiles in the three groups of children with ASD, ADHD, and ASD+ADHD and demonstrated the following findings (19, 20). In the study of Ashwood et al. (19), all children had a normal intellectual level representing a selected population and the combined group ASD+ADHD had the worst performance in adaptive evaluation with lowest scores among the three groups enrolled in this research. Further, it raised the question of whether intellectual abilities and social cognition are partly independent and act in different skill domains in ASD profiles. All the children with ASD+ADHD had a cognitive level in the normal range nonetheless demonstrated relevant adaptive difficulties supporting the hypothesis of distinct domains of neurodevelopment within the single child.

Also in the study by Turygin et al. (20), adaptive scores were lowest in the combined group, ASD+ADHD, and children with ASD resulted to be the more impaired among the three groups of study. However, this difference was not found to be significant between the combined and ASD group similar to the findings of the current study. Toddlers with co-occurring ASD+ADHD may represent a group that demonstrates greater early deficits in functioning compared to those with ASD that deserve further studies and follow-up monitoring. As to the cognitive level in the current study a higher range of intellectual abilities was found for the ADHD-only group, the other two groups presented IQ scores between the mean and borderline range of value (ASD: IQ about 81 and ASD+ADHD: IQ about 76). The wider range of IQ level in this sample represents more reliably the ASD population, in which differences in IQ scores are usually observed. Importantly it has been reported that children with ASD+ADHD with lower cognitive level have more severe social impairment, and greater delays in adaptive functioning than children with ASD only (39). In conclusion children with ASD+ADHD demonstrated a more severe adaptive impairment in comparison to children with ASD only even if not reaching statistic significance.

As an additional remark and a future direction in the evaluation of adaptive skills in ASD it is important to note that minimal clinically important differences (MCIDs) on VABS-II scores have not been rigorously established in ASD. To fill that gap a large multisite study has been carried out and lower VABS-II standardized score MCID estimates were observed for younger and more cognitively impaired children. This should be taken in account when evaluating adaptive functions in ASD concomitant to intellectual disability alone or combined with ADHD (40–42,).

There are some limitations in the current study that should be mentioned. Adaptive functions have been detailed in three different clinical groups, but the lack of a typically developing control group is the first limitation to be noted. A second limitation pertains to the relatively small sample size that may have influenced the between-group differences reported as to adaptive and cognitive ability and to the effect of IQ on adaptive function that may be underestimated. A higher number of participants would likely reflect more accurately these differences between the groups. Lastly this is a cross sectional evaluation of the three clinical groups and longitudinal studies of adaptive functioning in ASD+ADHD are strongly needed in order to increase the understanding of the development, change, and stability of symptoms over time and to identify protective and worsening factors of these conditions.

Children with ASD+ADHD had a greater treatment need which could imply additional treatments for both school and community services (26, 43, 44).

When symptoms are not managed, they may lead to more severe psychiatric comorbidity as well as poorer school, family, and cognitive outcomes among this population and so specific attention is warranted to readily provide appropriate intervention.

## Ethics Statement

The study is carried out in accordance with the recommendations of Good Clinical Practice (GCP) guidelines, with written informed consent from all subjects. All subjects gave written informed consent in accordance with the Declaration of Helsinki and its later supplements and local legal requirements. The IRB is the Institutional Review Board, at the University Hospital of Siena approved the study procedure and all study documents.

## Author Contributions

VS contributed to the design of the study, recruit and made multiple assessments of the children. FB, MS and AP contributed to the evaluation and recruitment of the children. LG participated in the design and the statistical analysis of the study. RC contributed in writing of the article and revised the methods of recruitment and selections of the study groups.

## Funding

No funding were received for this research.

## Conflict of Interest Statement

The authors declare that the research was conducted in the absence of any commercial or financial relationships that could be construed as a potential conflict of interest.
